# CXCL13 Promotes the Effect of Bone Marrow Mesenchymal Stem Cells (MSCs) on Tendon-Bone Healing in Rats and in C3HIOT1/2 Cells

**DOI:** 10.3390/ijms16023178

**Published:** 2015-01-30

**Authors:** Feng Tian, Xiang-Lu Ji, Wan-An Xiao, Bin Wang, Fei Wang

**Affiliations:** Department of Orthopaedic Surgery, Shengjing Hospital of China Medical University, Shenyang 110024, China; E-Mails: Xianglu_Ji@163.com (X.-L.J.); Wan_anXiao@163.com (W.-A.X.); Bin_Wang1011@163.com (B.W.); Fei_Wang1001@163.com (F.W.)

**Keywords:** marrow mesenchymal stem cells, rat, tendon-bone healing, CXCL13

## Abstract

Objectives: Mesenchymal stem cells (MSCs) are potential effective therapy for tissue repair and bone regeneration. In present study, the effects of CXC chemokine ligand-13 (CXCL13) were evaluated on tendon-bone healing of rats. Methods: Tendon bone healing of the rat model was established and biomechanical testing was performed at 2, 4, 8 weeks after surgery. Murine mesenchymal cell line (C3HIOT1/2 cells) was cultured. The expression of miRNA-23a was detected by real-time PCR. The protein expression of ERK1/2, JNK and p38 was detected by western blotting. MiR-23a mimic and inhibitor were used to overexpress or silence the expression of miR-23a. Results: MSCs significantly elevated the levels of ultimate load to failure, stiffness and stress in specimens of rats, the effects of which were enhanced by CXCL13. The expression of miR-23a was down-regulated and the protein of ERK1/2 level was up-regulated by CXCL13 treatment in both *in vivo* and *in vitro* experiments. ERK1/2 expression was elevated by overexpression of miR-23a and reduced by miR-23a inhibitor. Conclusions: These findings revealed that CXCL13 promoted the tendon-bone healing in rats with MSCs treatment, and implied that the activation of ERK1/2 via miR-23a was involved in the process of MSCs treated bone regeneration.

## 1. Introduction

Tendon-bone junction injuries are very common in sports. The surgical reattachment of tendon to bone often fails due to regeneration failure of the specialized tendon bone junction. Bone marrow-derived mesenchymal stem cells (MSCs) are multipotent adult stem cells and widely applied to organ repair and cell therapy [1,2], and the application of MSCs for tendon bone healing is reported with promising results [3]. MSCs can differentiate into osteoblasts, chondrocytes and adipocytes [4], and their osteogenic differentiation potential has been found in both *in vitro* and *in vivo* studies. MSCs can be directed induced to the injured sites and then play an important role in tissue regeneration.

As previously discussed, MSCs have the ability to differentiate into osteoblasts and adipocytes, which possibly be regulated by certain signals under injury conditions. It is reported that chemokines and their receptors are involved in the process of cell migration [5]. CXC chemokine ligand-13 (CXCL13) that binds monogamously to the CXC chemokine receptor-5 (CXCR5) is a key molecule involved in B-cell chemotaxis. Studies have found that inflammatory cytokine IL-6 significantly induced CXCL13 expression in human osteoblast cells but not osteoclasts [6], which implies that CXCL13 may play a role in the process of tendon-bone healing. However, much less is known about the role of CXCL13 in MSCs and no report has emerged to discuss the effect of its in tendon-bone healing.

MicroRNAs (miRNAs) have been regarded as key regulators in diverse biological pathways. Recent studies have shown that miRNAs contribute to pluripotency of stem cells [7,8]. It was reported that the reduced expression of miR-23a markedly increased TNF-α induced bone marrow MSCs apoptosis [9], but the overexpression of miR-23a is observed to promote the survival of MSCs exposed to hypoxia and serum deprivation [10]. In addition, it was demonstrated that miR-23a significantly impede osteoblast differentiation, and its effects can be reversed by the corresponding anti-miRNAs [11]. However, little is known about how miR-23a plays a role in bone regeneration.

Mitogen-activated protein kinase (MAPKs), including Extracellular signal-Regulated Kinase (ERK), c-Jun NH2-terminal Kinase (JNK) and p38, are the major signal transduction osteoblast molecules regulated by growth factors, cytokines, and stress, which consequently trigger long-term cellular responses [12]. It is well known that MAPKs regulates the differentiation of mesenchymal stem cell to the osteogenic or adipogenic lineage [13,14,15]. In the present study, we hypothesized that CXCL13/CXCR5 may play a role in the effects of MSCs on tendon-bone healing via miR-23a regulation. The tendon-bone healing model of rat was established to test this hypothesis and *in vitro* experiments were also employed to explore the mechanism underlying the effects of CXCL13.

## 2. Results and Discussion

### 2.1. Results

#### 2.1.1. Mesenchymal Stem Cells Differentiating into Osteoblasts and Adipocytes

**Figure 1:**
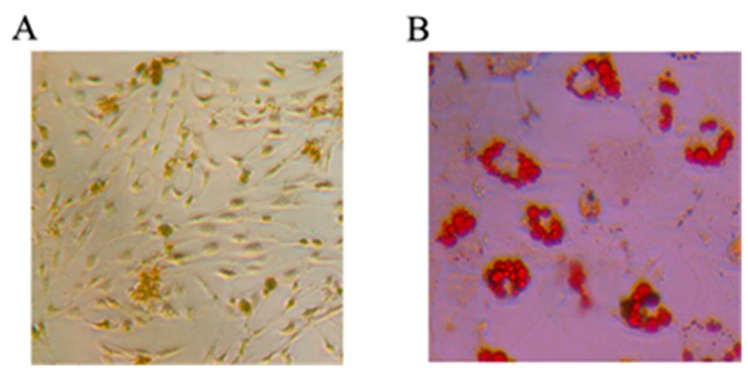
The potential of mesenchymal stem cells (MSCs) differentiating into osteoblasts and adipocytes. The Alizarin red staining of calcified nodules and Oil red staining were respectively applied to identify osteoblast differentiation ability (**A**) and adipogenic differentiation ability (**B**) of rat MSCs.

The MSCs were respectively cultured in osteogenesis and adipocytes induced medium and then differentiated for 14 days. Alizarin red staining of calcified nodules was applied to identify osteoblast differentiation ability of rat MSCs. Oil red staining was used to identify adipogenic differentiation ability. As shown in Figure 1, MSCs had the potential to differentiate into osteoblasts (Figure 1A) and adipogenic cells (Figure 1B).

#### 2.1.2. Biomechanical Assessment

**Figure 2:**
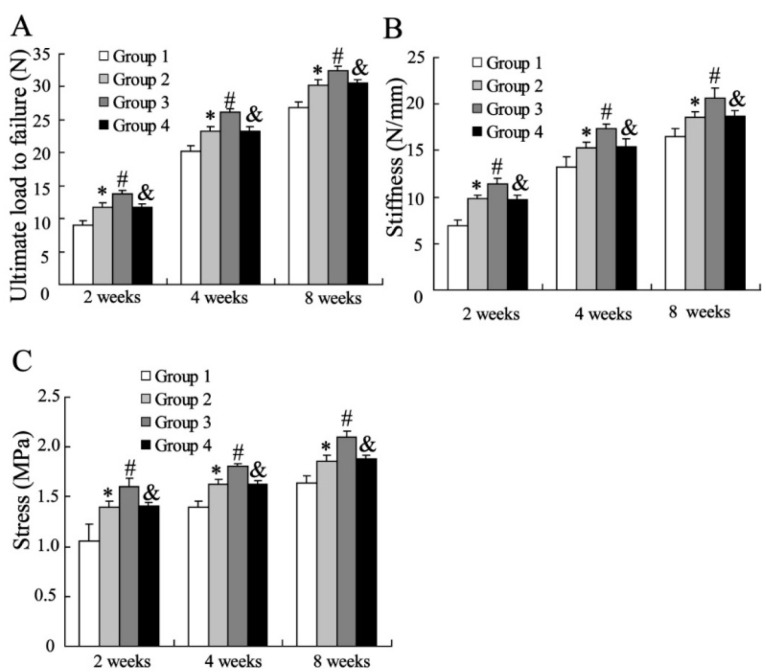
Biomechanical assessment of the tendon at the insertion site: (**A**) Ultimate load to failure; (**B**) Stiffness; (**C**) Stress. Data were shown as mean ± SD. * *vs.* Group1 (Injection with medium), *p* < 0.05; # *vs.* Group2 (Injection with MSCs), *p* < 0.05; and & *vs.* Group 3 (Injection with CXC chemokine ligand-13 (CXCL13) treated MSCs), *p* < 0.05. Group 4 is the specimens injected with CXCL13 treated MSCs accompanied by infection with lentiviruses of silencing CXCR5.

The biomechanical assessments were performed at 2, 4 and 8 weeks after surgery. As shown in Figure 2, MSCs significantly elevated the levels of ultimate load to failure, stiffness and stress in specimens of injured tendon-bone at 2 weeks. CXCL13 enhanced the effects of MSCs on all biomechanical markers of specimens, and of which was reversed by infection with lentiviruses of silencing CXCR5. In addition, the results were also observed at 4 and 8 weeks.

#### 2.1.3. The Effects of CXCL13 on miR-23a and MAPK Molecules Expression *in Vivo*

**Figure 3:**
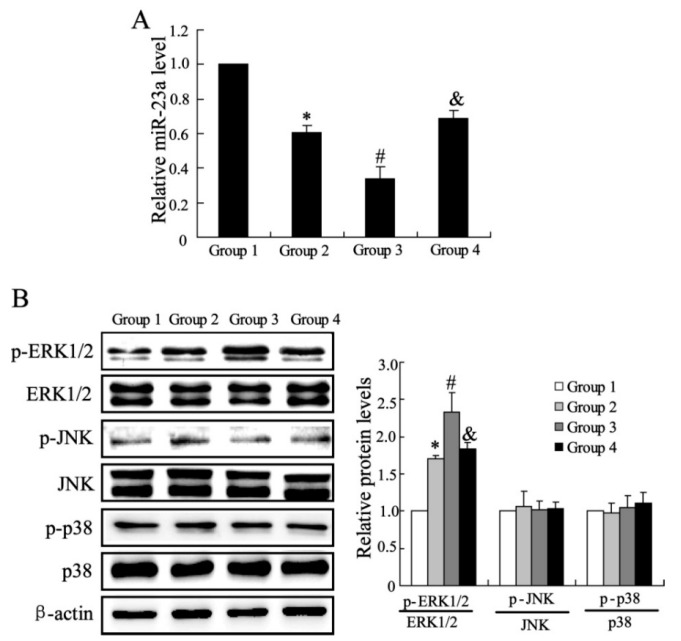
The effects of CXCL13 on miR-23a and mitogen-activated protein kinase (MAPK) molecules expression *in vivo.* (**A**) The miR-23a expression; (**B**) The protein expression of ERK1/2, JNK and p38. Data were shown as mean ± SD. * *vs.* Group1 (Injection with medium), *p* < 0.05; # *vs.* Group 2 (Injection with MSCs), *p* < 0.05; and & *vs.* Group3 (Injection with CXCL13 treated MSCs), *p* < 0.05. Group 4 is the specimens injected with CXCL13 treated MSCs accompanied by infection with lentiviruses of silencing CXCR5.

To identify the mechanisms underlying the effects of MSCs and CXCL13 on the tendon bone healing, the expression of miR-23a was detected by real time PCR. As shown in Figure 3A, the relative miR-23a level was significantly down-regulated by MSCs treatment. Compared with tendon in Group 2, the ones treated with CXCL13 and MSCs had a lower level of miR-23a expression. Interference of CXCL13 receptor canceled the effect of CXCL13 on miR-23a expression. The western blotting was also used to detect the protein expression in specimens. As described in Figure 3B, the total proteins of ERK1/2, JNK and p38 had no significant difference among four groups, and no difference was observed in phosphorylated JNK and p38 level. However, the MSCs markedly up-regulated the expression of p-ERK1/2, the effect was enhanced by CXCL13. Interference of CXCL13 receptor down-regulated the expression of p-ERK1/2 compared with group injection with CXCL13 treated MSCs.

#### 2.1.4. The Effects of CXCL13 on miR-23a and MAPK Molecules Expression *in Vitro*

**Figure 4:**
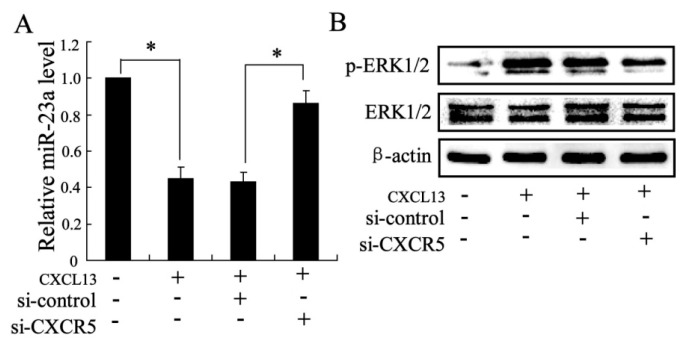
The effects of CXCL13 on miR-23a and MAPK molecules expression *in vitro*. (**A**) The miR-23a expression; (**B**) The protein expression of ERK1/2. Data were shown as mean ± SD; *t*-test of independent sample was used to analyze the difference between two groups; * considered as significant difference.

*In vitro* experiments were explored to examine the effects of CXCL13 on C3HIOT1/2 cells. As shown in Figure 4A, the CXCL13 significantly reduced the expression of miR-23a in cells, and si-CXCR5 abolished the CXCL13 induced down-regulation of miR-23a. Similarly, the protein expression p-ERK1/2 was up-regulated by CXCL13 treatment, and additional treatment with si-CXCR5 reduced the p-ERK1/2 level in cells.

#### 2.1.5. The Effect of miR-23a on ERK1/2 Expression *in Vitro*

**Figure 5:**
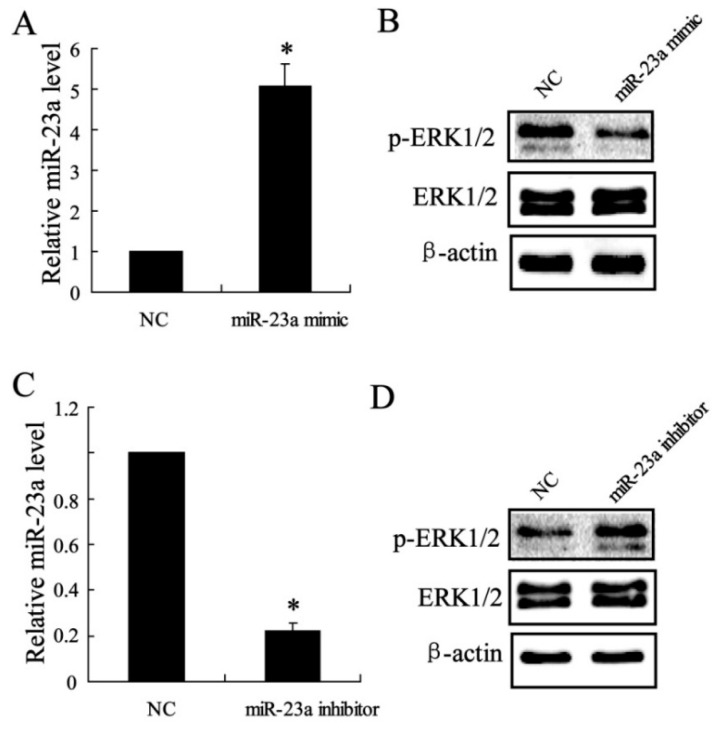
The effect of miR-23a on Extracellular signal-Regulated Kinase (ERK)1/2 expression *in vitro.* The relative miR-23a expression was quantified by real-time PCR (**A**,**C**); The p-ERK1/2 and ERK1/2 expression were detected by western blotting (**B**,**D**); Data were shown as mean ± SD; *t*-test of independent sample was used to analyze the difference between two groups; * considered as significant difference.

To further clarify the relationship between miR-23a and ERK1/2 in C3HIO1/2 cells, the expression of miR-23a was regulated by miR-23a mimic or miR-23a inhibitor. As shown in Figure 5A,C, the expression of miR-23a was effectively overexpressed or inhibited. The results in Figure 5B,D showed that p-ERK1/2 level was significantly down-regulated by miR-23a mimic and the expression was up-regulated by miR-23a inhibitor.

### 2.2. Discussion

In recent years, MSCs has been well known to be a promising therapy for tissue regeneration and bone healing [16,17]. However, the molecular mechanism underlying it has been still not fully understood. In present study, we demonstrated that chemotactic factor CXCL13 promoted the effect of MSCs on tendon-bone healing in rat experiment, and regulation of miR-23a and MAPK signal was involved in the process of tendon-bone healing.

Bone has a dramatic capacity for regeneration following injury, and the process is initiated by recruitment and differentiation of progenitor cells of mesenchymal origin along with inflammatory cells [18]. As previously reported, MSCs expressed numerous growth factors and chemotactic factors [19,20], which are crucial to modulate the activity of MSCs and promote the engraftment efficiency at damaged sites. CXCL13, belonging to the CXC chemokine family, is also known as B lymphocyte chemoattractant and plays its role by interacting with its specific receptor CXCR5. Lisignoli *et al.* [21] demonstrated that CXCL13 chemokine production by bone MSC was significantly elevated in osteoarthritis patients. Few studies have been emerged to discuss the effect of CXCL13 on tendon-bone healing. In present study, *in vivo* experiments confirmed that CXCL13 could promote the effects of MSCs on tendon-bone biomechanical assessment of rats following tendon-bone injury and the additional treatment of CXCR5 inhibitor reversed this effect.

To clarify the molecule mechanisms of CXCL13 involved in the promotion of tendon-bone healing, we investigated the effects of CXCL13 on the expression of miR-23a, which is found to suppress the bone regeneration [11]. The quantitative real-time PCR result showed that miR-23a expression was significantly down-regulated by MSCs injection, and CXCL13 treatment further promote the decrease of miR-23a expression in specimen. In addition, *in vitro* experiment also observed that CXCL13 reduced the level of miR-23a in C3HIO1/2 cells. These finds imply that inhibition of miR-23a is involved in the effects of CXCL13 on tendon-bone healing treated by MSCs.

MAPKs are key molecules to regulate the differentiation of MSCs to the osteogenic or adipogenic lineage [13,22]. In the present study, we detected the protein expression of ERK1/2, JNK and p38 in specimens of rats, and only elevated ERK1/2 expression was observed in MSCs treatment group compared with controls. ERK1/2 is an important member of the MAPK family of serine/threonine protein kinases [23]. The regulation of the ERK1/2 pathway has been reported to be the balance between the osteogenesis and adipogenesis of human adipose-derived stem cells [24], and plays an important role in the driving the extracellular matrix induced osteogenic differentiation of human MSCs [25]. In this study, we found ERK1/2 expression was up-regulated by CXCL13 treatment, the effect of which was reversed by si-CXCR5 in C3HIOT1/2 cells. In addition, we also demonstrated that miR-23a regulated the expression of ERK1/2. miR-23a overexpression down-regulated the phosphorylated ERK1/2 level and miR-23a inhibition up-regulated p-ERK1/2 level. These results suggest that CXCL13 plays a role in tendon-bone healing by ERK1/2 regulation via miR-23a. The present study implied that CXCL13 supplement in MSCs is a potential therapeutic strategy for promoting tissue regeneration and bone healing in clinic, which should be further studied in animal models and humans.

## 3. Experimental Section

### 3.1. Isolation and Culture of MSCs

All experiments were approved by the Animal Research Ethics Committee of the Shengjing Hospital of China Medical University. MSCs were isolated from the tibia and femur of 6 weeks old healthy, male Sprague-Dawley (SD) Rat rats, which were purchased from the Better Biotechnology Co., Ltd. (Nanjing, China). Pentobarbital (30 mg/kg) was administered intravenously to perform anesthesia. Bone marrow was washed and cultured in Dulbecco’s modified Eagle’s medium (DMEM) containing 10% fetal bovine serum (Gibco, Carlsbad, CA, USA). 1 × 10^6^ cells/mL was seeded in culture bottle and the medium was replaced after 72 h culture. The non-adherent cells were removed and the medium was replaced every three days. When cells were grown to 80% confluence, 0.25% trypsin was used to dissociate cells to monolayers. Seeded 5 × 10^4^/mL cells in culture bottle for three doublings and then injected into rats.

### 3.2. Tendon-Bone Healing Model of Rat

SD rats were anesthetized with an intraperitoneal injection of ketamine (80 mg/kg) and xylazine (5 mg/kg). Anesthesia was maintained using 1% to 1.5% isoflurane. A single dose of procaine penicillin (200,000 IU/kg) was given intramuscularly before surgery. A longitudinal incision was performed on the surface of the Achilles tendon and made it visualization. Separated the soft tissue, freed the Achilles tendon and cut off the stop point. The epiphysis was cleared and tendon was cut off 1 mm to make sure no stop points there. A 6-0 non-absorbable material needle was used to suture the tendon by Kessler technique. Proximal calcaneus performed a drilling with diameter of 0.8 mm and injected with 100 μL DMEM (Group 1) or MSCs (5 × 10^6^ cells, Group 2) or MSCs infected with lentviruses of overexpressing CXCL13 (Group 3) or MSCs infected with lentiviruses of overexpressing CXCL13 and silencing CXCR5 (Group 4).

### 3.3. Biomechanical Testing

Forty rats were sacrificed at 2, 4, and 8 weeks after surgery and allocated for biomechanical testing as previously described [26]. In brief, each specimen was preloaded to 0.1 N and then loaded to failure at a rate of 14 mm/s, corresponding to approximately 0.4% strain. The ultimate load to failure and the failure site were recorded. The displacement was measured using a 1-mm-resolution micrometer system attached to a linear stage. The linear region of the load-displacement curve was used to calculate the stiffness for each specimen. The ultimate stress at failure was calculated by dividing the ultimate load-to-failure by the cross-sectional area.

### 3.4. Cell Culture

Murine mesenchymal cell line C3HIOT1/2 cells were obtained from Shengjing Hospital of China Medical University. The cells were cultured in DMEM containing 10% fetal bovine serum, 50 Units/mL penicillin and 50 mg/mL streptomycin, and incubated in a humidified atmosphere at 37 °C with 5% CO_2_. The medium was changed every 3 days.

### 3.5. Real-Time PCR

MiR-23a was isolated from cells or tendon tissue by the TRIzol reagent (Takara, Dalian, China) and then performed a reverse transcription with specific RT primers. The level of miRNA was quantified by 7900HT Real-Time PCR System (Applied Biosystems, Foster City, CA, USA), and U6 small nuclear RNA served as an internal normalized reference.

### 3.6. Western Blotting

Protein extracts were extracted from C3HIOT1/2 cells or tendon specimen in rats. Equal amounts of protein were loaded onto sodium dodecyl sulfate-polyacrylamide gels (SDS-PAGE) and transferred to nitrocellulose membrane, which was blocked with 5% non-fat dry milk in Tris-HCl buffered saline incubated with the primary antibodies (Cell signaling, Danvers, MA, USA) according to the manufacturer’s recommendations. Immunoreactivity was detected by enhanced chemiluminescence (Millipore, Billerica, MA, USA). β-actin was served as a control protein to quantify the expression of target protein.

### 3.7. Overexpression and Down-Regulation of miR-23a

The expression of miR-23a in C3HIOT1/2 cells was down-regulated or overexpressed by transiently transfected with mimic or inhibitor using the Lipofectamine 2000 reagent (Invitrogen, Carlsbad, CA, USA) following the manufacturer’s instructions. The miR-23a inhibitor, miR-23a mimic and negative control were produced by RiboBio Co., Ltd. (Guangzhou, China).

### 3.8. Statistical Analysis

Results are expressed as mean ± SD. Differences among groups were compared with analysis of one-way ANOVA, followed by post-hoc test, using Dunnett’s method. *t*-test of independent sample was used to analyze the difference between two groups. *p* < 0.05 was considered statistically significant.

## 4. Conclusions

In conclusion, our study revealed that CXCL13 promoted the tendon-bone healing in rats with MSCs treatment. *In vitro* experiments suggested that the activation of ERK1/2 via miR-23a was involved in the process of MSCs treated bone regeneration. The present findings offer a theoretical basis for better application of MSCs to bone healing and imply that CXCL13 is a potential molecular target to bone regeneration.
